# Advances in Artificial Intelligence‐Based Liver‐Related Semantic Segmentation Techniques and Applications Using CT Imaging

**DOI:** 10.1002/cam4.71730

**Published:** 2026-04-19

**Authors:** Jun Pu, Xuan Wang, Liang Zhu, Jie Pan

**Affiliations:** ^1^ Department of Radiology Peking Union Medical College Hospital Beijing People's Republic of China

## Abstract

**Background and Aims:**

Artificial intelligence (AI)‐assisted semantic segmentation of liver computed tomography (CT) images has important clinical value in disease assessment, surgical planning, treatment evaluation, and longitudinal monitoring. This review aims to summarize the current clinical applications and recent technical advances in AI‐based liver‐related semantic segmentation on CT.

**Methods:**

This narrative review synthesizes recent studies on liver organ, tumor, and vascular segmentation, focusing on both clinical applications across different hepatic diseases and technical developments in model architectures, data processing, information fusion, and strategies for improving robustness and generalizability.

**Results:**

AI‐based segmentation models, particularly those built on U‐Net and hybrid attention/Transformer frameworks, have enabled automated analysis for clinically relevant tasks such as future liver remnant estimation, graft volumetry, chronic liver disease assessment, tumor burden evaluation, prognosis prediction, and vascular‐intervention planning. Despite strong performance in liver organ segmentation, challenges remain in small tumor and fine vascular segmentation, as well as in external validation, deployability, and workflow integration.

**Conclusions:**

AI‐based liver CT segmentation shows strong potential to support precision hepatobiliary imaging and to improve existing clinical workflows. Further progress will depend on improving robustness, reducing computational burden, enhancing performance in fine‐structure segmentation, and facilitating real‐world clinical deployment.

## Graphical abstract

This review summarizes clinical and technical advancements in AI‐based semantic segmentation for liver organ, tumors, and vasculature on CT imaging. It highlights key applications in surgical planning and disease evaluation while discussing critical challenges and strategies for real‐world clinical deployment and multimodal integration. Grouping chart of liver‐related segmentation.

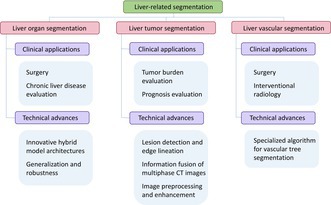

## 1 Introduction

The liver plays a critical role in numerous physiological processes and is frequently involved in both primary and secondary benign and malignant conditions. Computed tomography (CT) is currently the most intuitive imaging modality for assessing both the physiological structure and pathological alterations of the liver. It enables clear visualization of the hepatic parenchyma, vascular and biliary systems, various primary and secondary tumors, as well as morphological and density variations associated with chronic liver disease [1, 2, 3]. This establishes CT as one of the first‐line imaging techniques for the clinical evaluation of hepatic disorders.

Clinical evaluation of CT images requires clinicians to manually delineate hepatic cross‐sectional contours, internal anatomical structures, and pathological features across dozens to hundreds of sequential slices to reconstruct three‐dimensional liver anatomy and lesions. This labor‐intensive process demands substantial expertise and introduces interobserver variability. Follow‐up assessments compound these challenges by necessitating meticulous comparisons across serial examinations, further increasing time requirements and subjective interpretation risks. The growing emphasis on precision medicine in radiological evaluation, coupled with technological advances yielding higher‐resolution CT images, underscores the critical need for automated liver imaging analysis tools.

**FIGURE 1:**
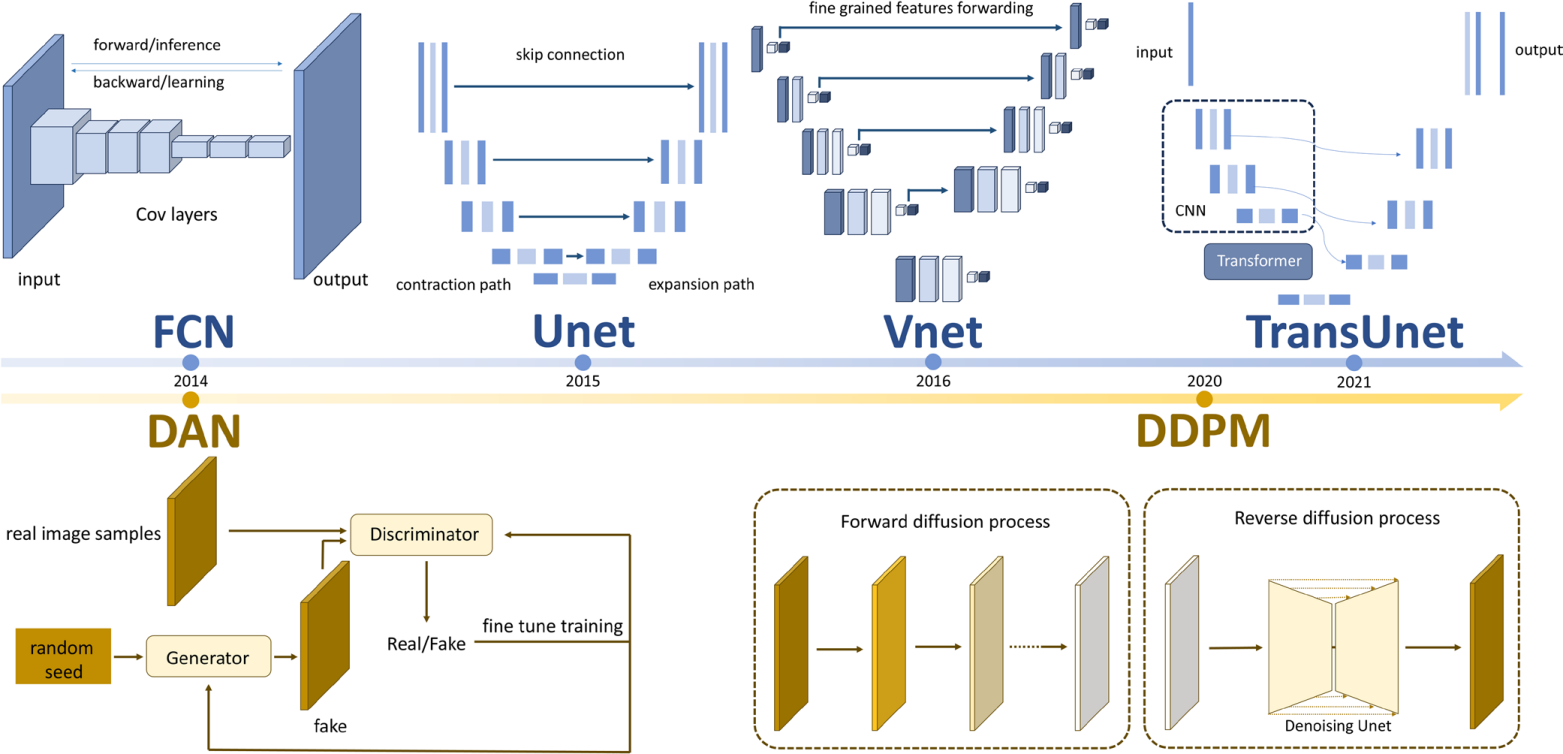
Development of deep learning semantic model architecture. DDPM, denoising diffusion probabilistic models; FCN, fully convolutional neural network; GAN, generative adversarial networks.

Recent advances in computer‐aided semantic segmentation techniques have significantly enhanced liver image analysis for clinical applications. Early methods relying on threshold‐based and region‐growing algorithms have evolved into deep learning approaches like Unet and Vision Transformer (ViT) [4], yielding not only improved segmentation accuracy but also enabling detailed delineation of fine anatomical structures. Since the introduction of fully convolutional networks (FCNs) in 2014, the architecture of deep learning‐based semantic segmentation algorithms has advanced rapidly in recent years. While novel variants continue to evolve from the U‐Net framework, emerging approaches such as generative adversarial networks (GANs) and diffusion models have also provided innovative perspectives for semantic segmentation (Figure 1). These developments in liver‐related semantic segmentation now permit precise identification of small tumors and intrahepatic vasculature, with important implications for clinical follow‐up, surgical planning, and chronic disease management [5, 6, 7].

## 2 Part 1 Liver Organ Segmentation

Liver organ segmentation constitutes a critical prerequisite for the precise delineation of intrahepatic pathological lesions and fine anatomical substructures. The macroscopic hepatic morphology, being directly correlated with preoperative assessment, surgical navigation, and longitudinal evaluation of chronic liver pathologies, underscores the pivotal role of liver organ segmentation in both biomedical research and clinical practice.

Manual segmentation of hepatic parenchyma on CT imaging remains labor‐intensive and time‐consuming, with inherent limitations in reproducibility and interobserver consistency due to subjective interpretation. These constraints have spurred the development of automated liver segmentation algorithms to enhance standardization and workflow efficiency. The release of open‐access liver segmentation datasets (Table 1) and the organization of associated challenge competitions [1] continue to attract artificial intelligence (AI) laboratories to explore state‐of‐the‐art semantic segmentation algorithms for liver organ segmentation. Positioning liver organ segmentation algorithms at the forefront of methodological innovation in liver‐related semantic segmentation research. In recent years, semantic segmentation algorithms for the liver have become increasingly robust. They now play a foundational role in various tasks, including the delineation of regions of interest (ROI) for liver tumor segmentation, the assessment of postoperative remnant liver volume, and risk stratification for populations at high risk of hepatic tumors.

**TABLE 1:** Commonly used datasets of liver and liver tumor images.

Dataset	Description
LiTS (liver tumor segmentation) [8]	Dataset containing 201 contrast‐enhanced abdominal CT scans with 3D segmentations of liver and liver tumors
TCGA‐LIHC [9]	Dataset containing CT, MRI and PET images of hepatocellular carcinoma patients without segmentation
3D‐IRCADB [10]	Dataset containing 30 3D abdominal CT scans with segmentations of liver organs and lesions
SLIVER07 [11]	Dataset containing 30 contrast‐enhanced abdominal CT scans with 3D segmentations of liver
CHAOS'19 [12]	Dataset containing CT and MRI images with 3D segmentations of liver, spleen and kidneys

Abbreviations: MRI, magnetic resonance imaging; PET, positron emission tomography.

### 2.1 Clinical Applications

#### 2.1.1 Surgical Applications

Surgery, including liver resection and transplantation, offers long‐term survival with good quality of life and is the only potential cure for large malignant tumor of liver [13]. Automated segmentation and volumetric calculation of the entire liver and its individual lobes and segments are critically important for preoperative assessment in liver tumor surgery. Postoperative liver function, which is largely determined by these volumetric parameters, is often a decisive determinant of eligibility for aggressive surgical intervention. In the context of major hepatectomy, a primary surgical modality for liver tumor, liver failure remains the leading cause of postoperative mortality. Consequently, the future liver remnant (FLR) serves as the primary predictor of postoperative hepatic failure. Xie et al. [14] trained multiple independent 3D U‐Net models using contrast‐enhanced CT images to separately segment the liver, Couinaud segments, hepatic veins, and intrahepatic portal veins. The total FLR volume and parenchymal volume calculated from these segmentations showed no statistically significant differences compared to manual assessments (*p* > 0.99 and *p* > 0.99, respectively). The study cohort included imaging data encompassing diverse pathologies, such as focal nodular hyperplasia (FNH), hepatic cysts, hepatic adenomas, hemangiomas, and hepatocellular carcinoma (HCC). The models demonstrated robust segmentation performance across these pathological backgrounds, achieving fully automated preoperative FLR prediction for major hepatectomy across a spectrum of liver diseases.

For patients with unresectable, localized liver malignancies meeting specific criteria, liver transplantation offers potential curative outcomes. Preoperative planning for liver transplantation similarly necessitates precise volumetric assessment of both the graft and the remnant liver to prevent large‐for‐size or small‐for‐size syndromes in recipients and to avoid insufficient remnant liver volume in donors. To accurately predict right lobe graft weight, Yang et al. [15] utilized delayed‐phase abdominal contrast‐enhanced CT images from 188 healthy subjects, with manual segmentation of the whole liver and right lobe, to train a deep learning model, UNETR. This model, comprising a Transformer‐based encoder and a convolutional neural network (CNN)‐based decoder, achieved automated segmentation of the whole liver and the right lobe. The Dice Similarity Coefficients (DSC) for these two segmentation tasks were 95.9 ± 1.0 and 92.4 ± 2.7, respectively, while the Hausdorff Distances (HD) were 5.2 ± 1.9 mm and 8.8 ± 2.9 mm, respectively. In a validation cohort of 20 preoperative liver transplantation cases, the predicted graft weights—derived using a calculation formula based on the segmented right lobe volume—showed no significant difference from intraoperative actual weights (*p* = 0.211), thereby achieving fully automated preoperative graft weight prediction. Namkee et al. [6] employed a 3D Residual U‐Net for left and right lobe segmentation, reporting DSCs of 0.94 ± 0.01 and 0.91 ± 0.02, respectively. The correlation between the segmented graft volume and the intraoperatively measured graft weight (*r* ^2^ = 0.76) outperformed that of manual segmentation performed by surgeons (*r* ^2^ = 0.68).

With advancements in AI recognition and segmentation technologies for fine hepatic structures, such as hepatic vessels, liver organ segmentation is evolving from gross organ contouring toward the segmentation of subanatomical structures, including liver lobes and segments. This evolution promises increasingly broad clinical utility in surgical contexts, providing anatomy‐based technical support for the intelligent optimization of perioperative management in liver tumor surgery.

#### 2.1.2 Chronic Liver Disease Evaluation

Chronic liver diseases, including cirrhosis, viral hepatitis, and metabolic dysfunction‐associated steatotic liver disease (MASLD), constitute significant risk factors for primary liver malignancies. Indeed, the progression from viral hepatitis/MASLD to cirrhosis and subsequently to HCC represents the predominant pathological trajectory for the majority of patients [16]. AI‐based semantic segmentation models enable the automated delineation of the liver, including its specific lobes and segments, while facilitating volumetric quantification and CT attenuation analysis. These capabilities establish a foundational framework for the automated comprehensive assessment of chronic liver diseases associated with hepatic tumors.

As patients with cirrhosis exhibit characteristic morphological changes, notably volume reduction in segments IV‐VIII with compensatory hypertrophy in segments I‐III, Lee et al. [17] developed a deep learning model for hepatic segment and spleen segmentation in contrast‐enhanced CT, achieving DSC > 91% for whole liver, spleen, and individual segments. The model‐derived liver segmental volume ratio (LSVR) (i.e., the volumes of Couinaud segments I–III/IV–VIII), whole liver volumes, spleen volumes showed strong correlation with manual measurements (*r* ^2^ > 0.80). The fully automated cirrhosis diagnostic model demonstrated area under the curve (AUCs) of 0.94 (training cohort) and 0.79 (test cohort), while the advanced fibrosis model achieved 0.80 and 0.71 respectively, indicating robust diagnostic performance.

Chronic hepatitis B virus (HBV) infection is one of the most significant etiological factors for hepatocellular carcinoma (HCC). Risk stratification in HBV patients is important for early intervention. Shin et al. [7] utilized DeepFore, a deep learning segmentation model of the abdominal organs, to compute a variety of CT imaging parameters including the liver volume, liver–spleen Hounsfield unit ratio, and established a fully automated liver cancer risk prediction model PLAN‐B‐DF in a training set of 4188 patients with hepatitis B with clinical indexes based on the gradient‐boosting machine, GBM algorithm. Based on the gradient‐boosting machine (GBM) algorithm combining the clinical indexes, an automated HCC risk prediction model PLAN‐B‐DF was established in a training set of 4188 hepatitis B patients. PLAN‐B‐DF can classify patients into four groups of minimal‐, low‐, intermediate‐, and high‐risk, with a 10‐year cumulative HCC incidence of 0.0%, 0.4%, 16.0%, and 46.2%, respectively (c index, 0.89). This model outperformed all previous conventional statistics (PAGE‐B, modified PAGE‐B, REACH‐B, CU‐HCC, GAG‐HCC, THRI, and PAGED‐B) and previous models that utilize only clinical and demographic variables in area under the precision‐recall curve (AUPRC) comparisons (*p* < 0.001). As this study exclusively involved Korean HBV carriers with predominantly genotype C infection, the model's generalizability to non‐East Asian populations and other HBV genotypes requires further validation.

The current clinical practice for imaging diagnosis of hepatic steatosis predominantly relies on manual region‐of‐interest (ROI) delineation to measure liver and spleen CT attenuation values. Yoo et al. [18] implemented a 3D nnU‐Net‐based liver‐spleen segmentation model to automatically generate organ masks, demonstrating excellent concordance between automatically derived CT attenuation values and those obtained through manual ROI delineation. Using magnetic resonance spectroscopy (MRS)‐proton density fat fraction (PDFF) as the reference standard, the study established a fully automated hepatic steatosis diagnostic system with severity grading in 362 liver transplant donors. The CT attenuation values and liver‐spleen attenuation differences derived from automated segmentation masks showed strong agreement with MRS‐PDFF (AUC = 0.813), achieving comparable diagnostic performance to manual ROI‐based methods. These findings validate the feasibility of CT‐based fully automated hepatic steatosis assessment systems.

The substantial population of patients with chronic diseases ensures abundant availability of imaging and clinical data, while the heavy workload of clinical evaluations drives urgent demands for developing automated assessment models. Early semantic segmentation models focusing on hepatic contours pioneered automated liver imaging analysis, establishing fundamental frameworks for quantitative evaluation. Contemporary advancements in increasingly sophisticated subanatomical structure segmentation and tissue component segmentation now provide multidimensional quantitative biomarkers—parameters unattainable through manual measurements—that enable comprehensive morphometric analysis of hepatic architecture. This technological evolution facilitates multidimensional characterization of chronic liver pathologies within current imaging technological constraints, offering novel analytical dimensions for precision hepatology.

The diagnosis and long‐term monitoring of patients with chronic liver disorders and associated tumors present a continuous clinical challenge. Automated diagnostic evaluation models can substantially alleviate repetitive clinical workloads. Early liver organ segmentation models established a foundation for developing automated assessment systems from liver imaging. For high‐risk populations, stratifying the risk of tumor development in patients with related chronic disorders is a central goal of these evaluations. Progressively refined segmentation of substructures and tissue components yields structural liver parameters that are difficult to obtain manually, thereby offering new approaches for individualized tumor risk stratification.

## 3 Technical Advances

### 3.1 Innovative Hybrid Model Architectures

Liver segmentation is the foundation for liver‐related semantic segmentation, delineating the working scope for algorithms that perform advanced tasks such as tumor segmentation. Consequently, as a pioneer in the application of novel algorithms for liver‐related semantic segmentation, it has fully undergone the entire process from early patch‐based classification algorithms to the current predominance of neural network architectures. Recent milestone developments in deep learning—particularly advancements in CNN architectures and attention mechanisms—have been systematically implemented and evaluated within liver segmentation frameworks. Architectural hybridization of diverse deep learning models has emerged as a critical research trajectory in hepatic semantic segmentation technology.

The CNN‐based classical medical image segmentation architecture U‐Net remains the most common underlying framework for liver organ segmentation algorithms, but it is prone to feature loss during encoding and poor global feature capture. The rapidly popularized attention mechanisms in recent years also face challenges in direct application to medical image semantic segmentation due to their massive parameter count and computational demands.

Li et al. [19] capitalized on the inherent strengths of attention mechanisms in modeling long‐range dependencies and spatial correlations across global contexts. By integrating an Attentive Context Encoding Module (ACEM) into a 2D CNN framework, they achieved segmentation performance comparable to the state‐of‐the‐art 2D‐3D hybrid method while maintaining favorable parameter efficiency. In a complementary strategy, Ou et al. [20] utilized a dual‐path approach employing parallel feature extraction through dedicated CNN and Transformer networks. This design attained a liver segmentation DSC of 95.35% on the LiTS2017 dataset while circumventing large parameter sizes and high computational complexity typical of pure Transformer models. Zhang et al. [21] further enhanced 3D U‐Net's capabilities by incorporating dual‐attention mechanisms—integrating position‐based and channel‐based feature extraction components—within the encoder. This modification elevated the model's liver segmentation DSC from 91.72% to 92.25% on the LiTS2017 dataset. Collectively, these architectural innovations demonstrate effective pathways for augmenting global feature representation in classical U‐Net frameworks without compromising computational practicality.

### 3.2 Generalization and Robustness

The varied nature of CT imaging—with its multiple scan phases and reconstruction methods—requires AI models to demonstrate strong adaptability and reliability in real‐world use. Meanwhile, the substantial computational demands and high parameterization inherent to large‐scale models create major obstacles for their practical implementation in clinical settings.

Ananda et al. [22] achieved superior multi‐phase CT liver segmentation without multi‐phase annotations through a dual‐discriminator based unsupervised domain adaptation (UDA) framework. By incorporating a boundary‐enhanced encoder to improve edge detection, their model outperformed state‐of‐the‐art methods including UDA, U‐Net + UDA, and ADVENT on different datacenters and different phases. Previous studies [23] demonstrated deep learning‐based CT image standardization to facilitate radiomics analysis across heterogeneous CT protocols and reconstruction kernels. However, reconstruction method variations (e.g., filtered back projection vs. iterative reconstruction) remain critical barriers for clinical deployment. Lee et al. [24] developed a GAN‐based normalization model that standardizes six reconstruction methods (Filtered Back Projection, Iterative Reconstruction, M40, M60, M80, Optical Projection Tomography) into M80‐equivalent images. When applied to 2D U‐Net segmentation, this standardization significantly improved DSC compared to native reconstructions (*p* < 0.001), establishing a practical pathway for reconstruction‐agnostic segmentation.

In clinical deployment of AI models, lightweight networks are commonly adopted to optimize computational efficiency and cost‐effectiveness, with cross‐domain capable architectures being particularly valuable for meeting multi‐task processing demands on shared clinical hardware. Qi et al. [25] developed a Generalizable Knowledge Distillation (GKD) framework that synergistically integrates dual technical innovations: Dual Contrastive Graph Distillation (DCGD) models the intracoupling and intercoupling semantic correlations, while Domain‐Invariant Cross Distillation (DICD) reconstructs domain‐invariant semantic vectors, achieving simultaneous generalization of lightweight networks hierarchically. This strategy achieved robust segmentation performance across heterogeneous medical imaging domains—including liver CT, retinal fundus photography, and colonoscopy video.

## 4 Part 2 Liver Tumor Segmentation

The diagnosis and evaluation of liver primary tumors and metastases is one of the most critical clinical applications of liver CT imaging. Due to the occultness and multifocality of primary and metastatic tumors and the dynamic changes of lesions throughout longitudinal care, hepatic tumor segmentation is both challenging and clinically valuable. An ideal liver tumor segmentation model could facilitate early diagnosis, revolutionize current follow‐up assessment paradigms, and contribute to surgical and ablation planning, thereby establishing an intelligent foundation for precision oncology in hepatic malignancies.

### 4.1 Clinical Applications

#### 4.1.1 Tumor Burden Evaluation

According to the Response Evaluation Criteria in Solid Tumors (RECIST) 1.1 guidelines for assessing therapeutic response in hepatic tumors, disease status (progressive disease [PD], partial response [PR], stable disease [SD], or complete response [CR]) is determined based on tumor burden evaluation using lesion diameter measurements. Due to the multifocal nature of hepatic tumors, clinical assessment entails substantial workload. Furthermore, indistinct lesion boundaries in raw imaging data may complicate target lesion selection and diameter measurement. Automated tumor segmentation could potentially reduce assessment difficulty while improving standardization and consistency of evaluation results. Joskowicz et al. [26] developed a liver tumor segmentation model using a simultaneous multi‐channel 3D R2U‐Net architecture, achieving a DSC of 0.82 for lesions > 5 mm in diameter. Following RECIST 1.1 criteria with 2 expert radiologists' evaluations as the gold standard, the researchers conducted a comparative study involving 86 abdominal CT scans from 43 patients. Two additional, different expert radiologists performed initial manual assessments, followed by reevaluations with computer‐assisted segmentation results after a 2‐week interval. The results demonstrated that computer‐assisted evaluation improved disease status classification accuracy in approximately 34.5% of cases.

Compared to the lesion selection and evaluation criteria specified by RECIST 1.1, total tumor volume may possess more profound clinical significance and prognostic value for colorectal cancer liver metastases (CRLM). However, manual segmentation of all lesions followed by volumetric calculation remains prohibitively labor‐intensive, rendering comprehensive volume assessment currently impractical. Wesdorp et al. [27] employed U‐net architecture to perform separate segmentation of liver parenchyma and CRLM, utilizing hepatic segmentation results as the target region for metastasis detection. The DSCs of liver and liver metastasis segmentation reached 0.96 and 0.86, respectively, and the intraclass correlation coefficient (ICC) of the training set and test set reached 0.97 and 0.98, respectively. Bereska et al. [28] developed a CRLM segmentation model using nnUNet architecture with subsequent volumetric analysis. The model attained DSCs of 0.87 and 0.85 for hepatic tumor segmentation in abdominal CT scans before and after neoadjuvant therapy, respectively. The ICC between total tumor volumes derived from manual and automatic segmentation reached 0.97.

#### 4.1.2 Prognosis Evaluation

To clarify the association between each parameter obtained based on automated liver tumor segmentation and patient prognosis, Keyl et al. [29] calculated metastasis surface area and volume using a CRLM segmentation model based on nnU‐Net, both of which were effective prognostic predictors (*p* = 0.035, *p* = 0.006). The multifactorial Cox proportional risk model consisting of liver metastasis surface area, abdominal muscle‐to‐bone ratio (MBR), and primary tumor location achieved a concordance index of 0.69 (95% CI: 0.65–0.72).

Xia et al. [5] conducted treatment response evaluation based on total tumor volume using a deep learning‐based liver metastasis segmentation model. The 3‐month disease progression assessment results demonstrated superior overall survival risk stratification capability compared to RECIST 1.1 criteria. These findings indicate that deep learning‐enabled volumetric treatment response assessment is not only efficient but also exhibits enhanced prognostic accuracy in response to patient outcomes when contrasted with conventional manual evaluation methods relying on maximum lesion diameter.

### 4.2 Technical Advances

#### 4.2.1 Lesion Detection and Edge Lineation

Conventional single CNN architectures demonstrate suboptimal performance in detecting small lesions. Al‐Battal et al. [30] proposed a dual‐Unet framework for liver tumor segmentation, wherein two distinct models were trained separately. The primary segmentation model, designed for general lesions, incorporates four downsampling and upsampling stages. In contrast, the auxiliary model for small lesion segmentation eliminates the final spatial reduction step and integrates a high‐resolution feature fusion module to incorporate high‐resolution stage features into skip connections of deeper stages of the model, thereby enhancing the detection and segmentation of small lesions. The optimal segmentation output was determined by comparing the features' separation between lesions and surrounding tissue. Li et al. [31] improved the Transformer architecture by employing hierarchical operations with varying receptive field sizes for different lesions, thereby enhancing feature learning capability for lesions of varied size, location, and morphology. Zhang et al. [32] modified the U‐Net architecture by introducing a multi‐scale adaptive feature fusion module to prevent the loss of small lesion information in high‐dimensional features. The integration of the attention mechanism further optimized the balance between global and local information.

Furthermore, due to the imbalance in imaging data across different pathological tumor types, models based on traditional loss functions exhibit faster convergence rates for common tumor types compared to rare ones, resulting in underfitting of the latter. Xie et al. [33] proposed Balance Dice Loss function to address the training data imbalance between primary and subsequent liver tumors.

#### 4.2.2 Information Fusion of Multiphase CT Images

Sun et al. [34] designed a fully convolutional neural network (FCN) with three specialized channels to extract and fuse arterial, portal venous, and delayed phase imaging features through higher‐layer integration. Their multi‐phase fused FCN (MC‐FCN) outperformed phase‐specific models (A‐FCN, PV‐FCN, and DL‐FCN) in segmenting lesions from the JDRD dataset. Liu et al. [36] designed a phase attention fusion (PA) module to assess feature weights for each phase at the voxel level, thereby enabling weighted fusion of multi‐phase image features. This modification increased the average DSC by 3.3% over standard nnUNet implementations. Due to registration issues between images acquired at different phases, Wu et al. [35] introduced a multi‐phase encoder to resolve interphase registration issues by extracting global and local cross‐phase features for ROI generation. A Transformer architecture then modeled inter‐ and intraphase ROI relationships, improving the DSC by 5.8%.

#### 4.2.3 Image Preprocessing and Enhancement

Liu et al. [37] attempted to improve noncontrast CT‐based image segmentation through pseudocolor image transformation. By applying pseudocolor conversion to enhance the contrast between hepatic tumors and background tissues, this image enhancement technique achieved dual benefits when applied to manual segmentation: a 50% reduction in processing time while improving segmentation accuracy. When implemented in mUnet‐based automatic segmentation, the DSC increased from 0.733 to 0.799. These results demonstrate that pseudocolor conversion can simultaneously optimize both conventional manual segmentation and artificial intelligence‐based automated segmentation methods.

Diao et al. [38] employed Gabor texture enhancement to screen texture differences between hepatic tumors and peri‐tumoral normal tissues. Considering that texture variations among different pathological types of hepatic tumors might affect the recognition and segmentation performance of automated models, the researchers automatically generated pseudo‐labels through clustering algorithms based on tumor texture characteristics. The constructed Texture‐based Auto Pseudo Label (TAPL) module demonstrated compatibility with multiple model architectures including Unet and mUnet, significantly improving the segmentation accuracy of existing models for hepatic tumors. This enhancement was particularly pronounced in the segmentation of small tumors with diameters ranging from 0 to 1 cm.

## 5 Part 3 Liver Vascular Segmentation

The hepatic vasculature serves as the anatomical basis for liver lobar and segmental division, as well as a critical consideration in surgical and ablation planning. Vascular anatomy‐based lobar segmentation represents the definitive morphological approach for liver parenchymal division. The strong correlation between clinical practices (including surgery and ablation) and hepatic vascular anatomy creates substantial demand for automated hepatic vessel segmentation. The intricate structure and anatomical continuity of vascular trees confer distinct characteristics in semantic segmentation tasks, while the parallel courses of hepatic arteries, portal veins, and bile ducts further increase the difficulty of their identification and segmentation. In recent years, various technical advancements in vascular tree segmentation have been applied to hepatic vascular segmentation models, demonstrating significant clinical application value.

### 5.1 Clinical Applications

#### 5.1.1 Surgery

Kazami et al. [39] trained a multi‐task 3D fully convolutional neural network using contrast‐enhanced CT images of 110 healthy liver transplant donors with manually annotated portal and hepatic veins. The model incorporated three decoders designed to: (1) extract all vascular structures, (2) identify center voxels within vascular regions, and (3) establish connections between center voxels, ultimately achieving comprehensive recognition, segmentation, and reconstruction of the portal and hepatic venous trees. The model demonstrated an error rate of 0%–4.4% in identifying portal vein branches from first‐ to fourth‐order divisions, with perfect accuracy (0% error) in classifying first‐order portal branches and the three main hepatic veins. These results significantly outperformed conventional tracking‐based algorithms. Researchers [40] further employed a 3D fully convolutional neural network to classify the identified portal veins into five branching clusters, achieving sector and segment delineation in combination with hepatic vein anatomy. The median volumes of AI‐automated segmentation results for the right hemiliver, each sector, and each segment showed no significant differences compared with manually validated ground truth values. This vascular segmentation‐based hepatic segment division model enables surgeons to preoperatively select target portal branches for transection, automatically delineate perfusion territories, and simulate postoperative remnant liver morphology and function, thereby providing crucial references for surgical planning. Zhang et al. [46] embedded hepatic vascular structures into both point clouds and voxel grids of the liver, achieving a 20% and 8% improvement in Dice scores for hepatic segment segmentation through complementary feature representation compared to state‐of‐the‐art point‐based and voxel‐based models, respectively. Oh et al. [6] developed a liver parenchyma and vascular segmentation model using a 3D residual U‐Net architecture for graft segmentation in CT imaging of liver transplantation donors. Clinical validation with 11 transplant cases demonstrated that the model‐estimated graft volumes showed significantly higher correlation with intraoperative measurements than manual estimations (*p* = 0.018). However, this study was limited by its exclusive use of healthy liver images for model training, making it difficult to assess the impact of pathological changes on segmentation performance. This limitation restricts the model's applicability beyond liver transplantation procedures.

#### 5.1.2 Interventional Radiology

**FIGURE 2:**
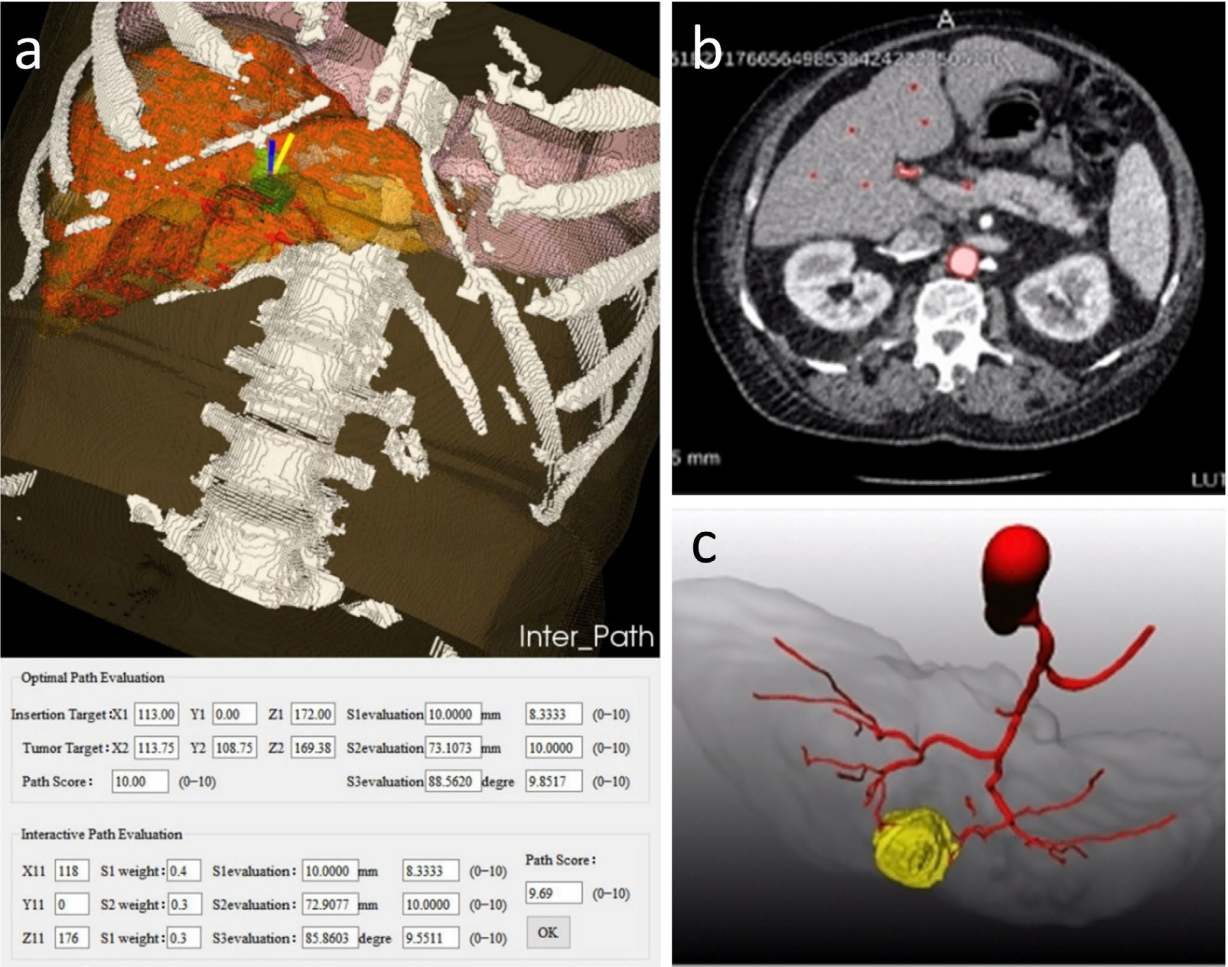
Surgical and interventional application of liver vascular segmentation. (a) Interactively adjusted interface of path‐planning system for interventional thermal ablation of liver tumors [41]. (b, c) Visual display of vessel segmentation for SIRT planning [47].

Song et al. [41] investigated automated planning of liver tumor ablation pathways based on automated hepatic vascular and tumor segmentation (Figure 2a). Incorporating hard constraints (e.g., avoiding critical structure contact, maintaining trajectory length below needle length) and soft constraints (e.g., maximizing distance from critical structures, minimizing path length) for needle trajectories following automatic segmentation of critical abdominal structures, optimal trajectories were selected through weighted scoring and Pareto optimality analysis. Among 18 automatically planned liver tumor ablation pathways, > 50% were deemed clinically usable without modification and 72% of planning requirements could be met when incorporating adjusted alternative pathways. This study represents a significant advancement in automated planning and standardized evaluation of ablation pathway based on segmentation.

To improve preoperative planning for selective internal radiation therapy (SIRT) in malignant tumors in the liver, Kock et al. [47] introduced a deep learning approach for hepatic artery segmentation. Their method utilized an aU‐Net, a modified 5‐level 3D U‐Net architecture, to train distinct segmentation models for fine hepatic artery branches and main trunks using 212 manually annotated arterial‐phase CT scans (Figure 2b). The final segmentation combined outputs from both models. The model demonstrated clinically viable segmentation accuracy for SIRT planning in 61.11% of cases (Figure 2c), outperforming conventional machine learning methods (25.71%).

### 5.2 Technical Advances

To accurately extract the subtle edge features of small blood vessels, Lim et al. [42] introduced a reverse attention module into TransUnet, which combines high‐level contextual information with low‐level features, achieving a DSC of 0.948 for hepatic vessel segmentation. The researchers also incorporated a Hounsfield unit (HU) window‐based image augmentation method, inspired by the clinical practice of adjusting window width and level to improve visualization. This approach enhanced the segmentation DSC by 3.4% compared to that obtained using the original images. Gao et al. [43] constructed a Laplacian salience filter to enhance vessel‐like regions while suppressing nonvascular areas. By combining this filter with a pyramidal feature extraction network, their method increased the DSC value by 1.63% over existing benchmarks on standard datasets. Tong et al. [44] achieved a 3.6% improvement in DSC for hepatic vein segmentation by incorporating a multi‐axial squeeze and excitation module (MASE) to confine excitation regions to hepatic vein territories, combined with a distribution correction module (DCM) to enhance learning of sparse spatial distribution features characteristic of hepatic veins. Xu et al. [45] implemented a multi‐task framework where the primary objective of mapping hepatic and portal veins was supplemented by two auxiliary tasks: centerline regression and vessel boundary segmentation. Their model attained a hepatic vein segmentation DSC of 0.824 and a portal vein segmentation DSC of 0.807, surpassing existing state‐of‐the‐art performance benchmarks.

## 6 Critical Synthesis and Discussion

Current research predominantly focuses on improving model segmentation performance, with state‐of‐the‐art results in metrics like DSC and HD95 serving as the primary outcome. However, the practical clinical deployment of these models—the translation from laboratory to bedside—receives far less attention. As indicated in Table 2, contemporary medical image segmentation algorithms, exemplified by 3D U‐Net and hybrid CNN‐Transformer methods, typically achieve a DSC above 0.95 for liver organ segmentation, where further metric gains offer diminishing clinical value. Consequently, model deployability and integration into clinical workflows are often overlooked. Some studies from a clinical application perspective omit or incompletely report critical deployment details, such as model parameters, hardware requirements, and inference latency [11, 12, 14]. Conversely, algorithm‐focused studies provide detailed metrics like parameter and floating point operations (FLOPs) counts [16, 17] but fail to contextualize them within specific clinical scenarios. Model parameters dictate the storage and memory demands of deployment hardware, while computational metrics like FLOPs or multiply‐accumulate operations (MACs) determine inference latency, directly affecting clinical utility. A standard U‐Net processing 512 × 512 inputs typically has around 30 million parameters and a computational cost of approximately 60 GFLOPs per inference. On servers with high‐performance graphics processing units (GPUs) like the NVIDIA RTX 3090 or A100, inference completes within tens of milliseconds. Various U‐Net derivatives, including Res‐UNet, HDense‐UNet, UNet++, 3D U‐Net, and V‐Net, have progressively increased in parameters and FLOPs relative to the classic U‐Net. The widely used 3D U‐Net for liver segmentation often requires minutes per inference on conventional hardware (Table 3). While acceptable for most radiology diagnostic workflows, this latency is inadequate for real‐time applications such as intraoperative navigation. The core self‐attention mechanism in Transformers has a computational complexity proportional to the square of the input sequence length (O(N^2^)), leading to significantly higher memory use and computational delays for 3D data compared to U‐Net and its variants, which impedes clinical adoption. As a representative hybrid architecture, TransUNet uses CNNs to extract local features and applies Transformers only on deep, low‐resolution feature maps. This design balances the capture of long‐range dependencies—such as vessel trajectories and organ positions—with computational complexity, thereby improving deployability. Nevertheless, such models often retain substantial parameter counts, which can be orders of magnitude larger than those of U‐Net [18]. For instance, the state‐of‐the‐art liver segmentation model ResTransUNet [20] achieves a computational complexity of 22.51 GFLOPs, among the lowest in mainstream models and only higher than U‐Net's 12.57 GFLOPs, yet its parameter count (53.75 M) remains over six times that of U‐Net (8.64 M). Although U‐Net and its variants still require performance improvements for tasks like hepatic vessel segmentation (Table 3), the self‐attention mechanism demonstrates high potential for such globally dependent tasks. Directly processing raw image data with non‐U‐Net architectures remains impractical under current hardware constraints. In contrast, the high plasticity of U‐Net allows for continuous architectural evolution through local innovations while maintaining manageable computational costs, cementing U‐Net and its variants as the dominant architecture in medical image segmentation. Amid rising model complexity, various compression and acceleration techniques—including quantization, pruning, and knowledge distillation—have been applied in medical image segmentation in recent years [48, 49, 50]. Lightweight models like LV‐UNet can reduce parameters and FLOPs by orders of magnitude compared to classic U‐Net [51], enabling real‐time segmentation in clinical settings. While most related work currently targets real‐time segmentation in ultrasound and endoscopy, techniques such as knowledge distillation are also emerging in liver segmentation studies [25].

**TABLE 2:** Liver organ segmentation on CT.

Study (references)	Architecture	Training dataset & setting	Segmentation task	Reported metrics (CT)			Clinical application	Notes on generalizability	Notes on deployability
				DSC	HD95 (mm)	MSD (mm)			
Yang et al. [15]	UNETR	Single‐center retrospective CT cohort of 188 living liver donor candidates	Whole liver	0.959 ± 0.001	5.2 ± 1.9	1.2 ± 0.4	Preoperative estimation of right lobe graft weight for living donor liver transplantation	Exclusion of patients with liver masses and cirrhosis	NR
			Right lobe	0.924 ± 0.027	8.8 ± 2.9	2.0 ± 0.7			
Xie et al. [14]	3D U‐Net	Single‐center retrospective CT cohort of 170 patient before major hepatectomy	Whole liver	0.984 ± 0.011	—	—	Automatic FLR assessment before major hepatectomy, supporting PHLF risk stratification	Patients with various liver conditions included in validation dataset	NR
			Couinaud's segments	0.94 ± 0.00	—	—			
			Hepatic mass	0.693 ± 0.247	—	—			
			Hepatic vein	0.66 ± 0.08	—	—			
			Intrahepatic portal vein	0.67 ± 0.07	—	—			
Lee et al. [17]	3D U‐Net	Single‐center retrospective CT cohort of 406 patients with hepatitis B	Whole liver	0.981 (0.978–0.985)	—	—	Noninvasive diagnosis of cirrhosis using liver–to–spleen volume ratio derived from automated segmentation	Lower performance in the nongenotype C HBV patients	Inference latency per CT: 33 s
			Spleen	0.957 (0.952–0.962)	—	—			Inference latency per CT: 12 s
Li et al. [19]	Attention‐based CNN	LiTS/3D‐IRCADb datasets	Whole liver (LiTS)	0.942	—	29.75	—	—	Higher segmentation accuracy with fewer parameters (49.13 M) than 3D DenseUNet
			Whole liver (3D‐IRCADb)	0.971	—	—			
Ou et al. [20]	Hybrid CNN‐Transformer method	LiTS/3D‐IRCADb/CHAOS/SLIVER07 datasets	Whole liver (LiTS)	0.953 ± 0.045	—	—	—	—	Intermediate parameters (53.75 M) between TransUNET and SwinUNet, with FLOPs (22.51G) lower than all but UNet among major models
			Whole liver (3D‐IRCADb)	0.964 ± 0.023	—	—			
			Whole liver (CHAOS)	0.954 ± 0.016	—	—			
			Whole liver (SLIVER07)	0.957 ± 0.180	—	—			
Zhang et al. [21]	Attention‐based CNN	LiTS dataset	Whole liver	0.926	—	28.09	—	—	NR

Abbreviations: DSC, Dice similarity coefficient; FLOPs, floating‐point operations; FLR, future liver remnant; HBV, hepatitis B virus; HD95, 95th percentile Hausdorff distance; MSD, mean surface distance; NR, not reported.

**TABLE 3:** Liver tumor and vascular segmentation on CT.

Study (references)	Target structure	Architecture	Training dataset & setting	Segmentation task	Reported metrics (CT)		Oncologic/Procedural task	Clinical performance	Notes on generalizability	Notes on deployability
					DSC	Sensitivity (Recall)				
Joskowicz et al. [26]	Liver metastases	3D UNet	218 clinical abdominal CT scans of 71 patients from two institutions	Lesions with diameter > 5 mm	0.82	0.86	Automated segmentation and registration of liver metastases in follow‐up scans to aid in disease status evaluation	Computer‐aided reading improved conventional reading in 34.5% cases	Two‐center training data of colon, breast, and pancreatic cancer patients, but predominantly hypodense lesions in the test data	Mean analysis time per pair: 54 ± 10 s
				Lesions with diameter > 10 mm	0.84	0.95				
Wesdorp et al. [27]	Liver metastases	UNet	434 single‐center CT scans	Unresectable CRLM (development cohort)	0.86	0.84	Automated segmentation and TTV assessing of CRLM	—	Poorer performance in external validation cohort with smaller median lesion diameter	NR
				Resectable CRLM (external validation cohort)	0.82	0.78				
Bereska et al. [28]	Liver metastases	nnUNet	Multi‐center cohort of 783 portal venous phase CT scans	CRLM (internal testing database)	0.85		Automated segmentation and TTV assessing of CRLM	—	Robust performance in pre‐ and postneoadjuvant therapy scans and external datasets	NR
				CRLM (external validation database)	0.83					
Keyl et al. [29]	Liver metastases	nnU‐Net	—	—	—	—	Automated segmentation of CRLM with calculation of total volume and surface area	Total surface area as an independent predictor of prognosis (HR 4.52, 95% CI:1.43–14.3, *p* = 0.01)	—	NR
Xia et al. [5]	Primary and metastatic liver tumors	Multi‐task learning architecture	Multi‐center cohort of 111 portal venous phase CT scans				Automated disease status assessment based on follow‐up scans	Superior prognostic performance of automated TTV assessment compared to RECIST 1.1	Inclusion limited to HCC and urothelial carcinoma liver metastases	NR
Kazami et al. [39]	Liver vasculature	3D FCN	Single‐center cohort of 110 donor candidates for living‐donor liver transplantation	PV	0.90 (0.68–1.0)		Support for virtual hepatectomy via vessel segmentation	Superior automatic recognition/extraction and reduced correction time vs. TA	Impact of large tumors/cirrhosis on vessel morphology not addressed	Significantly reduced correction time for misclassified/missing branches (9.2 min vs. 24.3 min with TA)
				HV	0.94 (0.81–1.0)					
Kazami et al. [40]	Couinaud segmentation based on vessel structure	3D FCN	Single‐center cohort of 174 donor candidates for living‐donor liver transplantation	Lobe	0.92–0.95		Automated delineation of perfusion territories (lobes/sectors/segments) based on vessel transection	High concordance between fully automated virtual hepatectomy and ground truth (matching rate: 92.8% for sectors, 91.6% for segments)	Lack of pathological liver images	Median time for fully automated segmentation: 1.4 min on a personal computer (Intel Corp., US, Core i7 with 16 gigabyte random access memory)
				Sector	0.86–0.92					
				Segment	0.71–0.89					
Oh et al. [6]	Section segmentation based on vessel structure	3D UNet	Single‐center cohort of 103 donor candidates for living‐donor liver transplantation	Portal vein	0.63 ± 0.12		Automated delineation of perfusion territories (lobes/sectors/segments) based on vessel transection	Higher correlation with intraoperative measurements compared to manual estimation	Lack of pathological liver images	NR
				Hepatic vein	0.74 ± 0.09					
				Lobe	0.91–0.94					
Song et al. [41]	Liver organ/vasculature/tumor	Existing 3D UNet					Path‐planning for interventional thermal ablation of liver tumors	78% of paths effective; efficiency superior to manual planning	Limited to liver tumors < 3 cm	Automatic planning: ~4 min; Interactive adjustment: ~4 min
Kock et al. [47]	Liver vasculature	3D UNet	212 hepatic arterial phase CT scans from two centers	Hepatic artery		0.653	preinterventional planning of SIRT	Outperformed traditional ML; sufficient for SIRT planning in 61% of cases (vs. 75% manual reference)	—	NR

Abbreviations: CRLM, colorectal liver metastasis; DSC, Dice similarity coefficient; HCC, hepatocellular carcinoma; HR, hazard ratio; HV, hepatic vein; ML, machine learning; NR, not reported; PV, portal vein; SIRT, selective internal radiation therapy; TA, tracking‐based algorithm; TTV, total tumor volume.

The heterogeneity of real‐world imaging data also challenges the clinical deployment of models. Variations in populations, scanning equipment, and reconstruction methods can degrade the segmentation performance of models predominantly trained on single‐center, homogeneous datasets. As Table 3 shows, the performance of several CRLM segmentation models declined in external validation cohorts [23, 24], and most studies in this review did not even establish such a cohort. Since the liver can harbor various primary and metastatic tumors, models based on a single tumor type are difficult to integrate into general clinical workflows, and their efficacy for other malignant liver metastases or primary liver tumors remains unproven. Some liver organ segmentation studies excluded patients with liver tumors or cirrhosis [15], while studies focusing on cirrhotic livers faced uncertainties regarding robustness in nongenotype C HBV patients due to single‐center bias [17]. Models trained on imaging from healthy liver donors, as included here, were not tested on data from patients with liver diseases [6, 36, 37]. Ideal medical image segmentation studies should incorporate imaging from patients with complex disease backgrounds in both training and testing. For instance, Xie et al. [14] constructed a training set of 1942 images from healthy, fatty, cirrhotic, and tumor‐bearing livers, and tested on 1399 images from another center, including cases of FNH, cysts, adenomas, hemangiomas, HCC, and cholangiocarcinoma. Sourcing data from CT scanners of different manufacturers endowed their model with high robustness, though it focused on liver segment rather than tumor segmentation. If these data were used for tumor segmentation, the volume for individual disease categories might still be insufficient. Establishing public imaging datasets encompassing multiple diseases and diverse populations is crucial for improving model generalization. Data platforms similar to the UK Biobank can provide important infrastructure for such research. Emerging electronic health platforms, such as China's Medical Insurance Imaging Cloud and the European Health Data Space, could, with proper consent and anonymization, become public datasets covering national or regional populations, providing reliable data for foundational model training. Until such ideal public datasets exist, multi‐center data remain vital for addressing homogeneous training data and limited volume. Federated learning can train multi‐center models without sharing original patient data, and personalized federated learning (pFL) can fine‐tune parameters for individual centers [49]. Domain generalization and adaptation, as mainstream methods to address distribution shifts, are widely applied in medical image segmentation [52]. Ananda et al. [22] also used such techniques to bridge feature differences across contrast phases, enabling a model trained on single‐phase data to perform multi‐phase segmentation. Standardizing heterogeneous data using GANs is another important approach; for example, Lee et al. used this method to improve a liver segmentation model's generalization across different reconstruction protocols [24]. Adjusting the loss function to increase the weight of data from specific disease categories can also mitigate representation bias and enhance real‐world robustness [33].

## 7 Beyond CT: Multimodal Synergies

Deep learning‐based semantic segmentation of the liver from MRI has advanced in parallel with CT‐based approaches, proving clinically valuable for tumor diagnosis, staging, treatment response assessment, preoperative planning, and fibrosis evaluation [53]. MRI offers unique clinical advantages over CT for liver imaging due to its lack of ionizing radiation, superior soft‐tissue contrast, and quantitative parameters like the proton density fat fraction (PDFF). Publicly available datasets now support this development; beyond the 120 MRI volumes in CHAOS, the 2023 Duke Liver Dataset (DLDS) provides 2146 abdominal MRI series from 105 patients, including 310 series with manually segmented liver masks [54]. Segmentation models for MRI must account for distinct challenges not present in CT, such as multi‐sequence protocols and the absence of a standardized intensity scale, necessitating multi‐channel architectures and more complex preprocessing. The direct transfer of algorithms between CT and MRI is further complicated by difficulties in cross‐modal image registration and inconsistencies in annotation standards [55, 56, 57, 58]. To address these generalization issues, techniques like domain adaptation and GAN‐based cross‐modal image synthesis have been employed for CT‐MRI fusion [55, 57, 58]. Integrating CT and MRI synergistically combines the geometric precision and clear depiction of bony structures from CT with the exceptional soft‐tissue contrast of MRI. Models leveraging this multimodal foundation enable more accurate segmentation and deliver comprehensive diagnostic, planning, and navigational information beyond the capability of any single modality [60].

## 8 Limitations and Future Directions

While current deep learning algorithms based on UNet and self‐attention mechanisms perform well in liver‐related segmentation tasks, achieving near‐perfect results for liver organ segmentation, their performance in segmenting small tumors and small vessels remains suboptimal. A key limitation in the field is the frequent focus on improving model performance without equal consideration for clinical deployability. Critical challenges for clinical translation include the hardware requirements for deployment, the constraints of inference latency in practical applications, seamless integration with Picture Archiving and Communication Systems (PACS) and Radiology Information Systems (RIS), and ensuring model generalization and robustness across diverse patient populations and scanning conditions. Future work should prioritize the segmentation of small tumors and fine hepatic structures, report model inference latency on standard clinical hardware, and address integration into specific clinical workflows. Research must also quantitatively evaluate performance differences between AI‐assisted and original workflows for specific clinical tasks, pursue multi‐center data for training, and emphasize external validation in complex disease contexts. Beyond the laboratory, the development of public databases should provide the necessary infrastructure for medicine in the artificial intelligence era. Furthermore, as AI models begin to enter clinical deployment, issues such as model interpretability, the formulation and refinement of regulatory and approval guidelines, and clinician training also warrant significant attention.

## 9 Conclusion

This review summarizes the clinical applications and algorithmic developments of AI‐based semantic segmentation models for the liver. These models provide substantial support in clinical scenarios such as liver tumor treatment evaluation, surgical planning, and chronic disease assessment, demonstrating the potential to modify existing clinical workflows. The UNet architecture continues to serve as the foundational framework for most mainstream models. Current model compression and acceleration techniques could be applied to reduce their parameter counts and computational complexity. Approaches like domain adaptation and federated learning could improve model generalization and robustness, while also enabling the multimodal fusion of CT and MR imaging. This paper further discusses common challenges—including computational complexity, generalization, and robustness—that currently limit the clinical deployment of these models and offers suggestions for future research.

## Author Contributions

Jun Pu and Xuan Wang contributed equally to the conceptualization, performed the literature search and review, designed the visualizations, and wrote the original draft of the manuscript. Liang Zhu and Jie Pan contributed to the conceptualization, supervised the project, and critically reviewed and edited the manuscript. All authors have read and agreed to the published version of the manuscript.

## Funding

This study was funded by Chinese Academy of Medical Sciences and Peking Union Medical College (CAMS & PUMC), Medical Imaging Big Data Analysis and Mining Platform: Assisted Disease Diagnosis and Precise Classification (A334000).

## Conflicts of Interest

The authors declare no conflicts of interest.

## Data Availability

Data sharing is not applicable to this article as no new datasets were created or analyzed during the current study. All data and information supporting the findings of this review are available within the article and the cited references.
